# Y-27632 enhances differentiation of blastocyst like cystic human embryoid bodies to endocrinologically active trophoblast cells on a biomimetic platform

**DOI:** 10.1186/1423-0127-16-88

**Published:** 2009-09-22

**Authors:** Kavitha Sivasubramaiyan, Swapnil Totey, Vijay Bhat, Satish M Totey, Kaushik Deb

**Affiliations:** 1 Stempeutics Research Pvt Ltd., Air Port Road, Bangalore KA 560017, India; 2 Stepeutics Research Malaysia Sdn Bhd, Kuala Lumpur, Malaysia; 3 Department of Biochemistry, Manipal Hospital Diagnostic Services, Bangalore KA 560017, India

## Abstract

Trophoblast differentiation and formation of the placenta are important events linked to post-implantation embryonic development. Models mimicking the biology of trophoblast differentiation in a post-implantation maternal microenvironment are needed for understanding disorders like placental-ischemia or for applications in drug-screening, and would help in overcoming the ethical impasse on using human embryos for such research. Here we attempt to create such a model by using embryoid bodies (EBs) and a biomimetic platform composed of a bilayer of fibronectin and gelatin on top of low-melting agarose. Using this model we test the hypothesis that cystic-EBs (day 30) that resemble blastocysts morphologically, are better sources as compared to noncytic EBs (day 10), for functional trophoblast differentiation; and that the Rho kinases inhibitor Y27632 can enhance this differentiation. Non/cytic EBs with/out Y27632 were grown on this platform for 28 days, and screened from secretion and expression of trophoblast and other lineage markers using ECLIA, RT-PCR, and Immunofluorescence. All EBs attached on this surface and rapidly proliferated into hCG and progesterone (P2) secreting functional trophoblast cells. However, the cells derived from cytic-EBs and cytic-EBs+ Y27632 showed the maximum secretion of these hormones and expressed IGF2, supporting our hypothesis. Also Y27632 reduced extraembryonic endoderm and trophoblast lineage differentiation from early noncystic-EBs, whereas, it specifically enhanced the induction of trophoblast and multinucleated syncitiotrophoblast differentiation from late cystic-EBs. *In vivo *trophoblast differentiation can be replicated in fibronectin based biomaterials, using cytic-EBs and by maneuvering the Rho-ROCK pathways. Response of EBs to a compound may vary temporally, and determination of their right stage is crucial for applications in directed-differentiation or drug-screening.

## Introduction

During preimplantation development, the embryos at the blastocyst stage form a polarized layer of outer epithelial cells called the trophectoderm [1]. Post-implantation this trophectoderm differentiate into mesenchymal like trophoblast cells [1]. The trophoblast cells invade through the maternal endometrium and proliferate in the maternal stromal cells while initiating a decidual reaction at the site of implantation [1]. The trophoblast cells are the precursors for the placenta that would develop latter [1]. Understanding the mechanism of placental cell differentiation and formation is important for developing interventions related to poor placental formations and birth defects. With a regenerative medicine point of view it would be fascinating to have placental cells growing in cultures, which could be employed for supporting pregnancies during medical conditions like placental ischemia or pre-eclampsia.

Human embryonic stem cells (hESCs) are known to have the ability to differentiate into embryonic and extraembryonic lineages, and have been used as models to understand lineage differentiation for a long time. They help in overcoming the ethical impasse on the use of human embryos for such research [2]. These cells are either subjected to directed differentiation on regular 2-dimentional cultures, or in suspension cultures, where they grow as 3-dimensional spheres known as embryoid bodies (EBs). These EBs mimic peri-implantation embryos in lineage composition, and are often used as the starting material for directed differentiation protocols. However, depending on the lineage induction time lines in these EBs may or may-not be suitable or more effective for deriving a specific lineage. We have therefore used EBs at two different stages; early EBs and late cystic-EBs (i.e., EBs which have developed a fluid filled cavity like that of a blastocyst) to compare their efficiency of trophoblast cell differentiation. Here we test the hypothesis that the cystic-EBs being morphologically similar to a blastocyst, may have an inherent ability to differentiate towards trophoblast cells and would be a better starting material for inducing such differentiations of extraembryonic trophoblast, or endoderm lineages. Human ESCs readily differentiate to placental precursor cells during in vitro culture [3,4]. However, many of the currently used differentiation protocols involve growth factors and conditions that have not been identified or found to be irrelevant to the differentiation process in vivo. Such differentiation protocols therefore limit our ability to understand the molecular mechanisms of placenta formation in vivo. We have therefore tried to develop a differentiation protocol based on Y27632 a commonly used inhibitor of the Rho -kinases, molecular players, which are known for their roles in trophectoderm and trophoblast formations in vivo.

Rho-family GTPases (such as RhoA, Rac1, and CDC42) regulate trophectoderm differentiation, cell polarity [5] and E-cadherin expression in cleavage stage embryos and a variety of other cell types [6]. Rho kinases (ROCK) are downstream effectors of the Rho GTPases. Inhibition of the ROCK activity can reduce or enhance cell polarity or differentiation in a dose dependent manner. Y27632 a known inhibitor of the Rho kinases (10 μM) can also improve human embryonic stem cell colonogenicity in small colonies [7]. The role of ROCK in differentiation of hESCs and the cells in the EBs is not entirely clear.

Here we have tried to mimic the embryonic developmental stages and the differentiation process by more than one way. First we have used cystic embryoid bodies which resemble blastocysts as a starting material for differentiation. These entities which are close to a late blastocyst in terms of having a similar fluid filled cyst/cavity, were found to be better than early EBs without any cysts. Second we have used a biomimetic platform which is rich in matrix proteins like fibronectin found in the endometrial stroma [8]. Third we have tried to derive and direct trophoblast differentiation by controlling the Rho-kinase signaling pathways which is well known for it role in differentiation. This model therefore would be useful to understand trophoblast differentiation and screen compounds for potential developmental toxicity.

## Materials and methods

### Regular culture of hESCs on MEFs and feeder free cultures on Matrigel

Human embryonic stem cell lines HUES7 and 9 were used after institutional ethics committee approval. The cultures were maintained as described in our previous publications [9].

### Induction of embryoid bodies (EBs)

For induction of embryoid body (EB) formation, the hESC were seeded on low-adherent 60 mm plate (BD Biosciences, San Jose, CA,) containing ES media without FGF2. Human ESCs from three confluent 35 mm dishes were collected after trypsinization and used for inducing EBs in each 60 mm low-adherent dishes. Culture media was changed every two days. Early EBs without any cavities was collected on day 10 of culture. Cytic EBs were collected on day 30 of culture.

### Selection of dose of Y27632 for hESC cultures

A commonly used dose of 20 μM Y27632was used for testing its effect on trophoblast differentiation. The compound was added to the regular culture media for cytic and non-cytic EBs after plating them on the biomimetic platforms.

### Preparation of the biomimetic platform

Low melting agarose (Sigma, St. Louis, MO) 0.05% was coated (0.1 mm thick) on 35 mm dishes, after 15 minutes at room temperature, this was then layered with a coating of gelatin 0.01% (Sigma, St. Louis, MO) in PBS. This was further coated by a top layer of Fibronectin (Sigma, St. Louis, MO) diluted to 0.005% using DMEM. 1 ml of this solution was added to the 35 mm culture dishes. The dishes were allowed to stay overnight at 25°C (room temperature). On the next day the fibronectin was aspirated and the plates were gently washed with the culture media twice. These platforms were equilibrated with DMEM overnight in a CO2 incubator before putting the EBs for outgrowth.

### Outgrowth of EBs into monolayers

Early noncystic and late cystic EBs were collected on days 10 and 30 respectively. They were washed in the culture media and were divided into groups of about 20 each. Each group was then grown in continuous presence of 20 μM Y27632, and allowed to attach and outgrow on our biomimetic platform. A control group of EBs (n = 20) was allowed to grow and attach without any Y27632 supplementation. The presence of all the germ lineages in the day 10 and 30 EBs were confirmed by RT-PCR analyses.

### Directed differentiation of the EBs to functional trophoblast cells

Day 10 and day 30 old EBs were collected and divided into treated and control groups. The treated EBs (n = 40) were grown in the continuous presence of 20 μM Y27632 over a period of 28 days. The control group (n = 40) was left unexposed to Y27632 and grown till 28 days. About 10 EBs were seeded in each of the biomimetic platforms for the control and treated groups. The rate of attachment of the EBs and their areas of outgrowth leading to an adherent monolayer of cells was carefully observed and monitored under a phase contrast inverted microscope (Nikon).

The culture media were changed every 4 days. 1 ml of the culture supernatants were collected from the control and treated culture dishes every 4 days from day 1 to day 28. The secretion of functional trophoblast specific hormones hCG and progesterone was measured using electrochemiluminescence immunoassay "ECLIA". Similarly the culture/spent media were also analyzed for Alfa feto protein (AFP) to detect signs of extraembryonic endoderm differentiation. The expression profile of some of the common pluripotency, ectoderm, endoderm, mesoderm and trophectoderm lineage markers like Oct4, Nanog, HMGB1, βIII-tubulin, GATA4, BMP2, Brachury, GCM1 and β-hCG etc. were studied by RT-PCR in the control and treated cells to confirm differentiation. These adherent monolayer of trophoblast cells were also studied for immunolocalization of early human trophectoderm and endoderm markers like SSEA1 and Cytokeratin-18. The trophoblast like characteristic of these cells was also confirmed by GCM1, Hand 1, IGF2 and GMC1 mRNA expression in the control and the Y27632-treated monolayers of cells.

### Electrochemiluminescence immunoassay "ECLIA"

The culture supernatants collected were tested for functional trophectoderm and endoderm differentiation by testing the presence of intact hCG+ β subunit, Progesteron II (P2), and AFP. The analyses were done using electrochemiluminescence immunoassay (ECLIA) for these molecules using Roche Elecsys Cobas diagnostic kits (Catalog numbers: 03271749, 12145383 and 04481798). The assays were carried out following the manufactures instructions, and were analyzed on the Roche Elecsys 2010 (Roche Diagnostics GmbH, Indianapolis, IN, USA) immunoassay analyzer.

### RT-PCR

Total RNA from cells was isolated using TRIZOL-LS Reagent (Invitrogen) as per the manufacturer's protocol. Complementary DNA was synthesized using the SuperScript III First-Strand Synthesis System (Invitrogen) as per the manufacturer's instructions. Polymerase Chain Reaction (PCR) was carried out using 1 U Taq DNA Polymerase (Sigma) and MgCl_2 _to a final concentration of 1.5 mM in a total volume of 25 μl/reaction. β-actin was used as the housekeeping control. PCR cycles consisted of an initial denaturation at 95°C for 5 minutes followed by 35 amplification cycles of denaturation at 94°C for 45 seconds, annealing for 45 seconds, and extension at 72°C for 45 seconds and final extension at 72°C for 10 minutes. The RT-PCR primers, amplicon sizes, and their annealing temperatures are given in Table 1.

**Table 1:** List of genes and RT-PCR primers used

Gene	Sequence	Annealing Temperature (°C)	Product Size(bp)
Oct 4	CGACCATCTGCCGCTTTGAG CCCCCTGTCCCCCATTCCTA	57	572

Nanog	CCTCCTCCATGGATCTGCTTATTCA CAGGTCTTCACCTGTTTGTAG	57	262

GAPDH	GGGCGCCTGGTCACCAGGGCTG GGGGCCATCCACAGTCTTCTG	60	531

βIIITubulin	CTTGGGGCCCTGGGCCTCCGA GGCTTCCTGCAGTGGTACACGGGCG	60	174

GATA4	TCCAAACCAGAAAACGGAAG CTGTGCCCGTAGTGAGATGA	60	187

Brachury	ACCCAGTTCATAGCGGTGAC ATGAGGATTTGCAGGTGGAC	60	216

BMP2	TGTATCGCAGGCACTCAGGTCAG AAGTCTGGTCACGGGGAAT	60	328

GATA2	TGACTTCTCCTGCATGCACT AGCCGGCACCTGTTGTGCAA	60	244

HAND1	TGCCTCAGAAAGAGAACCAG ATGGCAGGATGAACAAACAC	60	274

BMP4	GTCCTGCTAGGAGGCGCGAG GTTCTCCAGATGTTCTTTCG	60	339

βhCG	GCTACTGCCCCACCATGACC ATGGACTCGAAGCGCACATC	55	95

GCM1	CGGAAACGCTGTCCCAACT CCCTTCAGGCTCAATGAGACG	57	235

IGF2	CAATGGGGAAGTCGATGCTG CTTGGCGAGCACGTGAC	61	421

FGFR1	GGACTCTCCCATCACTCTGCAT CCCCTGTGCAATAGATGATGATC	56	109

### Immunofluorescence

EBs were grown on coverslips coated with the biomimetic platform as per requirement. The cells were fixed with 4% paraformaldehyde (Sigma) followed by permeabilization in 0.2% Triton X100 (Sigma). The slides were then incubated with primary antibodies 1:500 dilution of SSEA1 (Santa Cruz Biotechnology, CA, USA), and Cytokeratin 8/Troma1 (R&D Systems, Minneapolis, USA) overnight at 4°C. After washing thrice with PBS, fluorescein isothiocyanate/Texas red- labeled Secondary antibodies against the primary goat/rabbit/mouse were added as 1:500 dilutions and incubated for 2 hours. DAPI (Sigma) was used for nuclear staining and then washed with PBS. The negative controls were done without primary antibodies. Slides were mounted with DABCO (Sigma) and images were acquired using Nikon Eclipse 90 i microscope (Nikon Corporation, Japan) and Image-Pro Express software (Media Cybernetics, Inc, Silver Spring, MD).

### Statistical analysis

We have used Student's *t*-test (paired or unpaired) as appropriate, for calculation of statistical significance. Error bars on the graphs show standard deviation of three or more replicates (n = 3) or samples. Values were considered statistically significant only when *P *< 0.05, and highly significant when *P *< 0.005.

## Results

**Figure 1:**
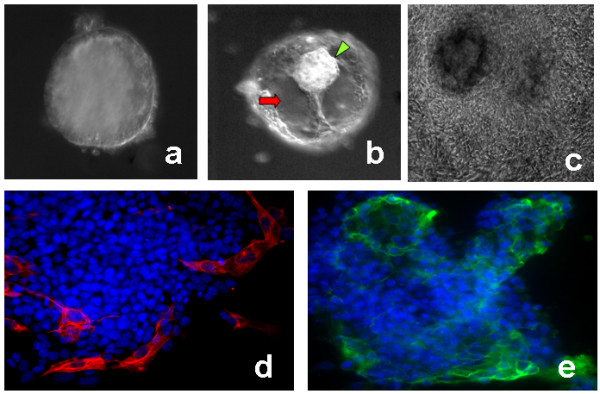
**Trophoblast differentiation from embryoid bodies on the biomimetic platform**. Panel (a) shows a non-cystic early EB. Panel (b) shows a late cytic EB with clear fluid filled cavity (red arrow) and a small mass of cells similar to inner cell mass (ICM) of an embryos (green arrowhead) pushed towards the top. Panel (c) shows a cystic EB outgrowing on the biomimetic platform on day 16. Panels (d) and (e) immunolocalization of trophoblast markers cytokeratin 8/Troma1 (red) and SSEA1 (green) in cytic EB outgrowths at early day 8. Blue (DAPI stain) represent the nuclei. This shows the evidences for the first signs for clear and distinct trophoblast cell differentiation from cystic EBs as early as day 8.

Embryoid bodies were made from hESC cultures grown feeder free for atleast one passage to get rid of any contaminating MEFs. We found that the early noncystic EBs (Fig 1a) and late cystic EBs (Fig 1b) showed a difference in their propensity to adhere to the designed biomimetic platform. About 80% of the early day 10 non cytic EBs attached to the platforms by day 2, the rest of them attached by day 4, and the first signs of cells spreading out were observed from this day. The late cytic EBs attached faster to these surfaces, and about 95% of them attached to this biomimetic surface by late day 1, the remaining cytic EBs attached and started outgrowing by day 3. The EBs from both groups when exposed to the continuous presence of 20 μM Y27632 showed enhanced and improved attachment and outgrowth rates. 20 μM Y27632 induced about 90% attachments in both the groups, by day 2. Fig. 1c shows an attached late cystic EB which has outgrown in presence of 20 μM Y27632.

### Secretion of hCG, P2 and AFP in noncystic EBs

**Figure 2:**
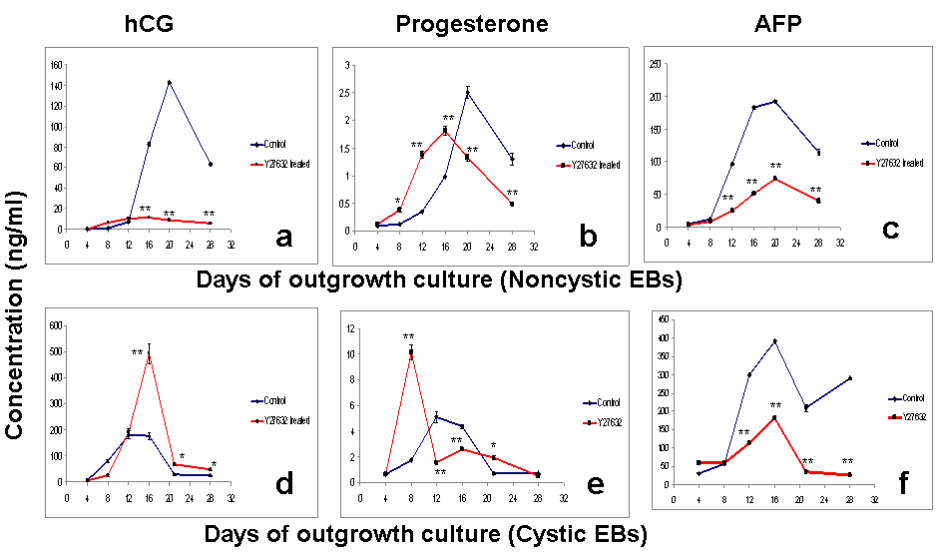
**Secretion of trophoblast and lineage markers**. Graphs (a), (b) and (c) indicates secretion of hCG, progesterone (P2) and AFP from control and Y27632 treated outgrowth cultures from non-cystic early day 10 EBs. Graphs (d), (e) and (f) indicates secretion of hCG, progesterone (P2) and AFP from control and Y27632 treated outgrowth cultures from cystic late day 30 EBs. The values are represented as mean ± SD. Level of secretion in the treated cultures are marked by (*) and (**) to indicate significant (P < 0.05) and highly significant (P < 0.005) differences as compared to the control.

The amount of functional extraembryonic lineage differentiation was measured by secretion of hCG and progesterone (trophoblast) and AFP (endoderm) in the culture supernatants. The early day 10 EBs showed high levels of secretion of hCG from day 12 onwards. The secretion of hCG peaked at day 20, and started falling after that (Fig. 2a). The 20 μM Y27632 treated cultures however, showed no such rise, and the secretion of hCG remained steady from day 8 to day 28 without any significant raise. These secretion levels were very significantly lower than the control levels on day 12 to 28. However, the secretion of hCG was triggered a bit earlier i.e., by day 8, in the 20 μM Y27632 treated samples, as compared to day 12 in the control.

The secretion of progesterone (P2) in the control cultures followed a similar pattern as that of hCG, with a steady raise in it levels from day 12 onwards (Fig. 2b). Also, the level of P2 steadily fell from day 20 to 28. We observed that 20 μM Y27632 treatment initiated the secretion of P2 earlier i.e., by day 8, as compared to day 12 in the control samples. P2 levels were significantly higher than the control in the 20 μM Y27632 treated samples up to day 16, and started falling from there. The highest levels of P2 secretion was again found at day 20 in the control samples.

The secretion of AFP was found to be very high from day 12 to 28 in the control as compared to the 20 μM Y27632 treated (Fig 2c). The highest level of AFP secretion was found on day 16 and 20 in the control. These were very significantly higher than that in the 20 μM Y27632 treated cultures.

### Secretion of hCG, P2 and AFP in cytic EBs

The cystic EBs were then checked for their potential to differentiate into functional extraembryonic trophoblast and endoderm lineages. Unlike the day 10 early noncytic EBs these EBs showed several hundred fold increase in the secretion of hCG both in the control and treated groups (Fig 2d). The secretion of hCG steadily raised from day 8, peaked at day12 and 16, and rapidly fell at day 20 in the control. Following this a steady secretion of hCG was maintained from day 20 t0 28. The 20 μM Y27632 treated samples exhibited a similar trend with several folds higher secretions on day 16, as compared to the control. The secretion levels in these 20 μM Y27632 treated cultures fell down significantly on day 20, and remained steady from day 20 to 28. However, the secretion levels of hCG in the 20 μM Y27632 treated cultures were significantly higher than the control from day 12 onwards.

The secreted levels of P2 in the control cultures followed a similar pattern as that of hCG in the control (Fig 2d and 2e). The peak secretion in the control cultures were seen on day 12, and was about 2 folds over the peak P2 levels obtained in the control cultures from noncystic EBs on day 20. The secretion of P2 was triggered early i.e., from day 4 onwards in the cystic EBs cultures treated with 20 μM Y27632. This effect is similar to that of in noncystic EB cultures treated with 20 μM Y27632. A sharp increase in P2 secretion was seen on day 8 in 20 μM Y27632 treated cultures. From day 12 to day 16 the level of P2 in the 20 μM Y27632 treated cultures remained significantly lower than the control. From day 20 to 28 the secretion of P2 in the control and treated cultures remained almost the same with no significant variations.

The secretion of AFP increased steadily from day 8 to 16 in both the treated and control groups (Fig 2f). The highest secretion was seen on day 16 in both the groups. The highest levels of AFP secretion from the cytic EBs were up by almost two folds as compared to that in non cystic early EBs (Fig 2c). The 20 μM Y27632 treated groups had significantly lower levels of AFP secretion as compared to the control. The levels of AFP decreased significantly from day 16 to 20 in both groups. While the group treated with 20 μM Y27632 maintained a very reduced levels of AFP, the control group showed a significant raise in AFP secretion again by day 28.

### Characterization of the trophoblast cells

The cells were characterized in the 20 μM Y27632 treated cultures of cystic EBs as early as day 8 i.e., from the day these cells started showing hCG and P2 secretions. RT-PCR analysis showed the expression of all the lineage and pluripotency markers with highest expression for the trophoblast markers likeGMC1, HAND1 etc. Immunofluorescence analysis of the trophoblast markers SSEA1 and Cytokeratin 8/Troma1 was also done. The EBs clearly showed cells positively for distinct surface localized SSEA1 and cytoplasmic and peripheral Troma1 in the cells surrounding the outgrowing cytic EBs (Fig 1d and 1e). Noncystic EBs did not show distinct expression for these markers on day 8 (data not shown). The immunolocalization of Cytokertin 8 also indicated that some of these trophoblast cells were multinucleated and were probably advancing towards the development of sincytiotrophoblasts.

**Figure 3:**
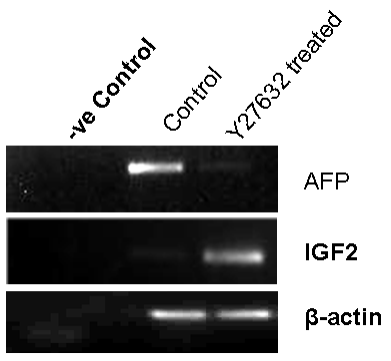
**Semiquantitative RT-PCR comparing the level of expression of AFP and IGF2 mRNA in control and Y27632 treated cytic EB cultures on day 16**. The expression of AFP was seen to be stronger in the control (very faint band) as compared to treated cultures. However, expression of IGF2 a characteristic of trophoblast cells was seen to be stronger in the treated as compared to the control (faint band). The expression of β-actin was similar in both control and treated samples.

To confirm that Y27632 downregulated AFP expression we tested the expression of AFP mRNA in the control and Y27632 treated cultures from cytic EBs on day 12 of outgrowth. We found that the expression of AFP was almost negligible in the Y27632 treated samples, and showed a strong band in the control samples (Fig 3). The levels of β-actin expression in both samples were found to be similar.

Another interesting feature of a functional post implantation trophoblast is the expression of IGF2 [10]. The expression of IGF2 was therefore tested in the day 12 samples of control and treated cultures. We found strong expression of IGF2 mRNA levels in the Y27632 treated samples as compared to the control (Fig 3). As described above the amounts of β-actin expression in both the samples were found to be similar and shows the semiquantitative nature of these estimations.

## Discussion

Human embryonic stem cell lines can differentiate spontaneously or directed towards trophoblast lineages by culturing them in presence of BMP4 [3]. Feeder free hESC cultures on matrigel in presence of mouse embryonic fibroblast conditioned medium (MEF-CM) and basic fibroblast growth factor (FGF2) readily respond to BMP family members by differentiating into trophoblast cells within a few days. Alternatively, trophoblast differentiation can be obtained from embryoid bodies inserted into matrigel rafts and cultured in absence of FGF2 and MEF-CM for up to 8 weeks (wk) [4]. A common factor in the two major protocols, developed so far, resulting in pronounced trophoblast cell differentiation was the inclusion of Matrigel, a complex extracellular matrix rich in laminin and other undefined regulatory molecules like TGFβ etc. Matrigel also lacks the stromal milieu needed to provide a stromal environment. Also no evidence for the role of BMPs in TB differentiation *in vivo *in embryos is known so far. Use of human embryonic stem cell (hESC) as a model to study the molecular players underpinning TB differentiation is therefore presently limited by the use of 1) undefined culture microenvironment for TB differentiation, 2) growth factors which are not yet shown to be implicated in trophectoderm differentiation during embryogenesis and 3) use of either human ESCs or EBs which may not be the right starting material that mimic perimplantation embryonic development.

In this study we have attempted to devise a more defined culture environment by providing a porous surface coated with known biological matrix. Gelatin a derivative of collagen and fibronectin are the components of the maternal stromal microenvironment. To have a defined culture condition the differentiation of EBs to trophoblast cells were done in culture media supplemented with knock out serum [11]. The uterine decidual or stromal cells provide an environment for growth and proliferation of the invading trophoblast cells of the implanting embryos [8]. Fibronectin has been implicated as a major player mediating the maternal fetal interactions and facilitate the trophoblast invasion [8]. The embryoid bodies attached better on our fibronectin coated surfaces (data not shown), as compared to normal tissue culture dishes, however, no signs of invasion into the gel was seen.

Presence of 20 μM Y27632 increased the rate of attachment and differentiation of trophoblast differentiation from the hESCs. Y27632 inhibits the Rho A, Rho Kinases, MLC kinase pathway, and activate the alternative CDC42 and Rac pathways. These pathways are also involved in maintaining pluripotency in hESCs [12]. These molecules are well known for their role in trophoblast cell migration and in determining cell polarity and in epithelial mesenchymal transitions [13]. The rate of attachment and the area of outgrowth were significantly higher in the Y27632 treated samples. The treated cells secreted higher amounts of hCG and P2 in the culture media. RT-PCR studies showed a reduced or loss of expression of the germ lineage markers (data not shown). However, a positive expression of trophoblast markers like hCG and IGF2 was found. This study for the first time indicates that Y27632 can be used for the induction of endocrinologically active syncitiotrophoblast like cells in a defined culture microenvironment. This also leads to the development of a model where trophoblast differentiation and proliferation is controlled by a known factor which plays a similar role in embryonic development [14].

It was equally important for us to have the right hESC based starting material that would be similar in lineage composition and characteristics of a post implantation embryo. This is essential for the model to reflect the *in vivo *developmental events closely. HESCs and their EBs are known to have different lineage compositions at different time points. Early stage EBs are composed of cells from all the three germ layers [2]. However, the localization and distribution of these germ cells in these three dimensional structures are not well studied across various hESC lines. We have seen that late EBs on the other hand have structures which are morphjologically closer to a preimplantation blastocyst. These EBs often known as the cytic EBs have a fluid filled blastocoel like cavity and also have a mass of cells resembling the inner cell mass pushed to on side (Fig 1b). Our study showed that cytic EBs produced more trophoblast lineage cells than early noncytic EBs. The fact that Y27632 can exert differential effects on early noncytic and late cystic EBs, indicates clearly that differentiation protocols, developmental studies, and drug screening protocols using EBs need to be designed keeping their temporally changing behavior in mind. I an earlier study we have shown that lipopolysaccharide (LPS) exposure could lead to a permanent silencing of mesoderm lineage induction in early EBs, by further screening the EBs at very late time points, we confirmed that this molecule did not simply delay the induction of mesoderm lineage in the EBs [2].

However, the potential of the hESC derived trophoblast cells to give rise to other placental cell types like the villious and extravillous TB cells remains to be tested. We have seen that the cells harvested from day 8 or 12 of outgrowth from cytic EBs can be expanded on fibronectin and gelatin coated plates for a few passages. We are now trying to see if trophoblast stem cells lines and other placental cells could be transdifferentiated from these primary cells. Our study clearly shows that the time points with highest hCG and P2 secretions may indicate the time when the cells should be harvested and taken to the next passage for deriving a trophoblast stem cell line. Such attempts are currently being undertaken in our laboratory.

## Conclusion

Understanding the molecular mechanisms of TB differentiation in embryos or from hESCs will help in devising better processes for directing differentiation of hESCs towards trophoblast and other placental lineages. Development of better biomaterial based three dimensional porous scaffolds micro-environments that mimic the endometrial stromal microenvironment, where trophoblast cells would invade into the biomimetic material and proliferate, is required. It would be important to use fibronectin, collagen1 and other stromal matrices to develop such platforms. Such advances will help in developing drug screening platforms that would also help in answering questions that are currently limited by the ethical impasse on using human embryos.

## Competing interests

The authors declare that they have no competing interests.

## Authors' contributions

KS and ST carried out the molecular genetic studies. VB carried out the immunoassays. SMT participated in the design of the study and revised the manuscript. KD conceived the study, carried out the ESC culture work, designed the experiments, performed the statistical analysis and coordination and drafted the manuscript. All authors read and approved the final manuscript.
